# The State of the Psychological Contract, Justice and Engagement Drive Nurses’ Performance Behaviors

**DOI:** 10.3390/ijerph192013505

**Published:** 2022-10-19

**Authors:** John Rodwell, Dianne Johnson

**Affiliations:** 1 Department of Management & Marketing, Swinburne University of Technology, Hawthorn, VIC 3122, Australia; 2 Griffith Business School, Griffith University, Brisbane, QLD 4111, Australia

**Keywords:** nurses, psychological contract, organizational justice, engagement, organizational citizenship behaviors, performance

## Abstract

This paper investigates the links between the psychological contract and organizational justice variables on to performance behaviors through the mechanisms of engagement, job satisfaction and psychological distress, beyond the perception-oriented individual factor of negative affectivity. Nursing staff (*n* = 273) from a medium to large Australian hospital completed a self-report survey. Structural equation modeling found differential effects of psychological contract breach and psychological contract status, the mediating roles of engagement, job satisfaction and distress on to performance behaviors, while noting the role of individual negative affectivity. Engaging nurses is critical to both their in-role and discretionary performance behaviors. Reducing negative impacts, particularly those due to breaking promises and unfair processes, while protecting the nurses’ mental health, enables performance levels to be maintained. This study demonstrates that nurses’ general perceptions of their employment relationship impacted their in-role and discretionary performance behaviors, especially through the mechanism of engagement. The complexity of managing nurses is highlighted by those variables that enacted positive impacts via engagement as versus the variables that led to distress and acted as brakes on performance, as well as the impact of the negative affectivity trait of the nurses.

## 1. Introduction

Nurses are under increasing pressure in the face of a shortage of staff and increasing demands associated with the growing prevalence of chronic disease and the growing heath care needs of an ageing population [1], pressures that have been exacerbated with the COVID-19 pandemic [2]. Retaining nurses in health care in the context of these pressures entails that organisations and their employment policies facilitate the retention of nurses and their provision of high quality health care [2].

One key approach for assessing nurses’ perceptions of their employment is through investigating the state of the nurses’ psychological contract with their employer. Qualitative research of nurses going through organizational restructuring found issues such as the nurses’ psychological contract and how fairly they were treated impacted their willingness to “go the extra mile” for the organization ([3], p. 66).

The willingness of nurses to perform activities beyond their job role, known as organizational citizenship behaviors (OCBs), is particularly important because nurses have the most frequent interactions with patients and nurses’ behaviors strongly influence the quality of care provided [4]. However, what are the mechanisms by which these behaviors are enhanced and what are the drivers of those mechanisms? Distinguishing whether the OCB is benefiting the organization (OCBO), or individual (OCBI) employees could help clarify these relationships [5]. Recent studies have found that key mechanisms driving nurses’ OCBs and in-role behavior (IRB) were their levels of engagement [6] and nurses’ perceptions of psychological contract breach negatively relates to outcomes such as job satisfaction, work engagement [7] and health issues [8]. Consequently, this study examines variables summarizing the employment relationship (psychological contract and organizational justice) and work performance behaviors (OCBO, OCBI and IRB) through the mechanisms of engagement, job satisfaction and psychological distress, beyond the role of perception-oriented individual factors, such as negative affectivity.

### 1.1. The Status of the Psychological Contract and Its Breach

The state of a nurse’s psychological contract is a useful summary of the state of the employment relationship, which can be understood as a social exchange based on a norm of reciprocity [9,10]. In this social exchange, as the employer fulfills promised obligations nurses are more likely to increase feelings of obligations to reciprocate [11,12]. These feelings of obligations may then lead to enhanced work performance, and possibly even performance beyond one’s formal job role [13].

Previous approaches to the psychological contract have often assumed that the employee values each of the elements of promises and obligations (e.g., pay, training) of the psychological contract equally, despite suggestions that such equal weighting is unlikely [14]. Accounting for the potentially varying levels of importance for each element allows for a better depiction of the general status of the psychological contract (PC Status). More specifically, PC Status can be characterized by the nurse’s overall perception of whether their employer is over-, just-, or under-fulfilling the psychological contract, along a continuum, considering the importance of each obligation respectively. The importance-weighted approach reflects a trade-off between the over-fulfillment and under-fulfillment of different issues [14]. Conversely, if one aspect of the psychological contract is highly important to an individual and not fulfilled, over-fulfillment in other areas may not prevent the ill effects of a poor psychological contract.

Yet, when the psychological contract has been broken (PC Breach), that breach may make unique contributions to nurses’ exchange relationship [8,10]. The processes involved in the impact of PC Breach are likely to be similar in some respects to PC Status yet different in important ways. For example, PC Breach could reflect less resources being available to nurses [15] to enable them to engage and cope at work, such as removing training or support from the organization, leading to lower engagement and higher distress. However, a key difference in the impact of breach relative to psychological contract fulfillment is that breach is likely to primarily and directly elicit an affective response (e.g., as anxiety and psychological distress), more so than a cognitive one [16].

### 1.2. Organizational Justice

A further key component of the employment relationship is how fairly employees are treated, known as organizational justice, which accounts for additional variance with outcomes such as mental health (e.g., [17]). There are four types of organizational justice: procedural, distributive, interpersonal and informational [18]. Procedural justice refers to the perceived fairness of decision-making processes used to determine outcome distributions such as pay and other rewards, as well as the openness of the organization to the input from nurses. Distributive justice pertains to the perceived fairness of the actual distribution of such outcomes. Interpersonal justice refers to perceptions of sincerity and respect in interacting with nurses. Finally, informational justice refers to the perceived adequacy and truth of explanations and information provided by the employer [18].

Most studies on justice examine only one or two justice types, or with interpersonal and informational justice types amalgamated into interactional justice despite evidence that they are distinct [18]. Thus it is unclear which types of justice are most applicable in a given situation, particularly as nurses pay attention to a range of fairness elements. For example, during a restructure nurses variously noted how good their communication was with their manager in terms of ‘distance’ (interpersonal), being unable to attend meetings (procedural) and whether they were given clear information, known as informational justice [3]. To clarify the nature of the potential relationships with various outcomes all four types of justice need to be investigated [10].

### 1.3. Potential Mechanisms on to Performance Behaviors

Several processes may be mechanisms linking the psychological contract to performance behaviors, including engagement, job satisfaction and mental health. Engagement can be important for all three forms of performance behaviors (IRB, OCBI and OCBO) for nurses across a variety of contexts [6]. Engaged employees are cognitively, emotionally and physically ‘present’, are invested in their work tasks and use more energy in their work [19]. Thus, from a social exchange perspective, the process may be that high PC Status may instill the nurse with a sense of owing the organization, where they respond to over-fulfillment of the psychological contract by ‘over-engaging’ to balance the exchange relationship, with increased motivation and personal energy invested into work [11].

Job satisfaction may also mediate the impact of the psychological contract on employee performance with previous research demonstrating relationships between psychological contract fulfillment, breach and job satisfaction [12,16]. Similarly, PC Breach negatively relates to nurses’ work related outcomes such as job satisfaction and engagement [7]. However, psychological contract research rarely examines psychological health, despite calls to do so [8,17]. With the consideration of psychological distress in this study, possible avenues for achieving high performance and low distress simultaneously, may generate options for nurse managers to work toward a state of productive wellbeing or sustainable performance.

In a similar manner to the psychological contract variables, organizational justice also has links to these outcomes. For example, positive links have been found between procedural, distributive and interactional (an amalgamation of interpersonal and informational) justice with higher job satisfaction [20]. Further, specific justice types link to psychological distress for nurses, such as procedural and informational justice [10]. Other studies have found positive links between types of justice and engagement, such as procedural, distributive and interactional justice [21]. However, there is limited research examining the interconnected relationships between the psychological contract and these outcome and performance variables.

### 1.4. Negative Affectivity

The psychological contract and organizational justice are fundamentally perceptual and therefore it is important to consider individual differences that may influence such perceptions. In particular, negative affectivity is a trait that can heavily impact perceptions, whereby those with high negative affectivity have an overall negative emotionality and tend to perceive themselves and the world more negatively [22]. However, despite empirical and theoretical links between negative affectivity, the psychological contract and justice, considering negative affectivity with these variables has been relatively uncommon [14], despite calls to include negative worldview traits in studies of nurses’ stress and job satisfaction [23].

### 1.5. Current Study

The current study addresses gaps in the literature surrounding the roles of various employment relationship variables (PC Status, PC Breach and four types of organizational justice) predicting work performance behaviors (OCBI, OCBO and IRB) in a large hospital, after accounting for negative affectivity. The study investigates possible mechanisms underlying the impact of the predictors onto performance in terms of engagement, job satisfaction and psychological distress.

Structural equation modeling found differential effects of psychological contract breach and psychological contract status, the mediating roles of engagement, job satisfaction and distress on to performance behaviors, while noting the role of individual negative affectivity. This study demonstrates that nurses’ general perceptions of their employment relationship impacted their in-role and discretionary performance behaviors, especially through the mechanism of engagement. The complexity of managing nurses is highlighted by those variables that enacted positive impacts via engagement as versus the variables that led to distress and acted as brakes on performance, as well as the impact of the negative affectivity trait of the nurses.

## 2. Materials and Methods

### 2.1. Participants

A total of 771 nurses and midwives from a medium to large hospital in Australia were invited to complete a survey. The paper survey was delivered via the internal mail system. A reply-paid envelope was provided so that completed surveys could be returned to the research team. Two hundred and seventy three responded to the survey (response rate 35.4%), all of whom were female. The majority of respondents had worked for the organization for five years or more (63.6%). Most of the respondents were employed on a part-time basis (60.1%) and many had completed tertiary studies (the highest level of education was most commonly an undergraduate degree for 36.9% of respondents or a postgraduate degree for a further 49.6%). The survey was comprised of previously validated measures, with most also tested in a nursing context (e.g., for the justice measures: [10]). The survey was approved by the Ethics Committee at Deakin University and then of the Australian Catholic University EC-206V and as extended.

### 2.2. Survey Measures

The PC Status measure was based on the seven common characteristics (e.g., ‘promotion and advancement’, ‘long term job security’ and ‘career development’) of the psychological contract proposed by Robinson [24], which in turn were drawn from Rousseau [25], weighted by importance using a process similar to that of Turnley and Feldman [14]. These seven characteristics were scored three times. The extent to which the characteristics had been promised and then fulfilled were assessed using the questions and rating anchors from Robinson [24]. That is, participants were asked to ‘Please indicate the extent to which your organization owes you, based on an implicit or explicit promise or understanding, for’ each of the seven characteristics, scored on a five-point rating (1 = not at all obligated, 5 = very obligated). Participants then indicated the extent to which the organization had fulfilled their promises for the same seven characteristics (1 = not at all fulfilled, 5 = very fulfilled). The participants then indicated each characteristic’s degree of importance to them personally, per the wording from Turnley and Feldman [14], although the scoring was from 1 = not at all important, to 5 = very important, rather than from 1 to 10, in keeping with the scaling of the Robinson [24] items.

For each of the seven characteristics, respectively, a weighted status score was calculated by subtracting the fulfillment score from the promised score, with the result multiplied by the characteristic’s importance score. The seven status values were then summed to make the overall PC Status measure. High scores were representative of a better PC Status, whereas low scores represented a poor PC Status. A similar index approach has been previously used to assess “the state of the psychological contract” [26].

PC Breach. Five items measured the general degree of perceived PC Breach developed by Robinson and Morrison [27] from 1 = strongly disagree to 5 = strongly agree. An example item is ‘almost all promises made by my employer when I started have been kept so far’.

Organizational justice was measured using a 20-item scale developed by Colquitt [18]. The scale consisted of four subscales and participants indicated the extent they experience each item relating to types of fairness on a five-point rating (1 = very often; 5 = rarely). Procedural justice was measured using seven items, for example ‘have those procedures upheld ethical and moral standards?’ Distributive justice was measured with four items. An example of an item in this scale is ‘Do your pay, promotions and other benefits reflect the effort you have put into your work?’ Interpersonal justice was measured using four items relating to people who made the decisions regarding participants’ pay increases and/or promotions, including ‘have they treated you with dignity?’ Informational justice was measured with five items, including ‘have they explained the procedures thoroughly’.

Negative affectivity was measured using a short five-item version [28] of the negative affect subscale from the *Positive and Negative Affectivity Scale* (PANAS; [22]). Participants rated the frequency with which they experienced each item (e.g., ‘nervous’, ‘scared’, ‘guilty’) over the past week on a five-point rating (1 = very slightly or not at all, 5 = very much).

Nurse’s engagement with their work was measured using a 12-item scale developed and validated by May, Gilson and Harter [29]. The extent that participants agreed with each item (e.g., ‘performing my job is so absorbing that I forget about everything else’) on a five-point rating (1 = strongly disagree, 5 = strongly agree).

Psychological distress was measured using the K-10, which has demonstrated good psychometric properties (e.g., [30]). Participants indicated how often they experienced each of the 10 items related to their psychological health in the last 30 days on a five-point rating (1 = all of the time, 5 = none of the time). Items included ‘did you feel tired for no good reason?’

The scale developed and tested by Agho, Price and Mueller [31] was used to measure job satisfaction. Participants rated the degree that they agreed with six statements relating to their job satisfaction on a five-point rating (1 = strongly disagree, 5 = strongly agree). Items included ‘I find real enjoyment in my job’ and ‘I like my job better than the average person’.

Performance behaviors, including organizational citizenship behavior. OCB was measured using a scale developed by Williams and Anderson [5] and validated by Rodwell et al. [6] consisting of three subscales with each item scored on a seven-point rating (1 = strongly disagree, 7 = strongly agree). Items 1–7 measured OCBI, with items such as ‘I help others who have been absent’ and ‘I go out of my way to help new employees’. Items 8–14 measured OCBO, with items such as ‘My attendance at work is above the norm’ and ‘I take undeserved work breaks’ (reverse-scored). Items 15–21 measured in-role behavior (IRB), with items such as ‘I fulfill the responsibilities specified in my job description’, ‘I meet the formal performance requirements of the job’ and ‘I perform the tasks expected of me’.

### 2.3. Analysis Approach

Structural equation modeling with maximum-likelihood estimation was used and several measures of model fit were adopted. Namely, the chi-squared (χ^2^) was used, a commonly used test of model fit, whereby a good fitting model is indicated by a small χ ^2^ and non-significant *p* value. Other tests of model fit were the standardized root mean square residual (SRMR), root mean square error of approximation (RMSEA), and comparative fit index (CFI). Models are considered to fit the data well when the following criteria are met: *p* > 0.05, χ^2^/df < 5, SRMR < 0.08, RMSEA < 0.06, TLI > 0.95 and CFI > 0.95 [32]. Initially, the data were checked for missing values, outliers and violations of normality. Cases that were missing more than a third of responses for items in any subscale were removed from the dataset leaving 234 cases. Further analyses revealed that data was missing completely at random. The remaining missing data were replaced using the maximum likelihood method.

The PC Status variable was a manifest variable in the model due to the method of its computation. Item parceling was used for the other variables applying the technique and formulae detailed and justified in Rodwell et al. [6]. Congeneric confirmatory factor analyses were conducted on the remaining variables, establishing unidimensionality for each of the parceled variables and providing evidence for discriminant and convergent validity through discrimination from the other variables, following Hoyle [32]. Consequently, a five-item global engagement factor was created using the five highest loading items, reflecting the more global nature of the variables studied here and the use of engagement as a higher order factor (per [33]). The two negatively-worded items from the PC Breach scale, the three IRB items reflecting rules adherence and three items from the justice scale and one OCBI item ‘I pass along information to co-workers’ were excluded from the model due to poor factor loadings and poor discriminant validity.

## 3. Results

The means, standard deviations, reliability coefficients and correlations are in Table 1. Note that the Cronbach alpha coefficients are provided on the diagonal of the correlation matrix and are in brackets in the table. The Cronbach alpha reliability coefficients range from 0.96 for PC Breach through to 0.83 for negative affectivity, down to 0.66 for OCBO in the table, with the reliability for IRB, of 0.97, being noted in the footnote of Table 1. Cronbach’s alpha was not obtained for PC Status because that is a calculated, manifest variable (effectively having an alpha of 1.00).

**Figure 1:**
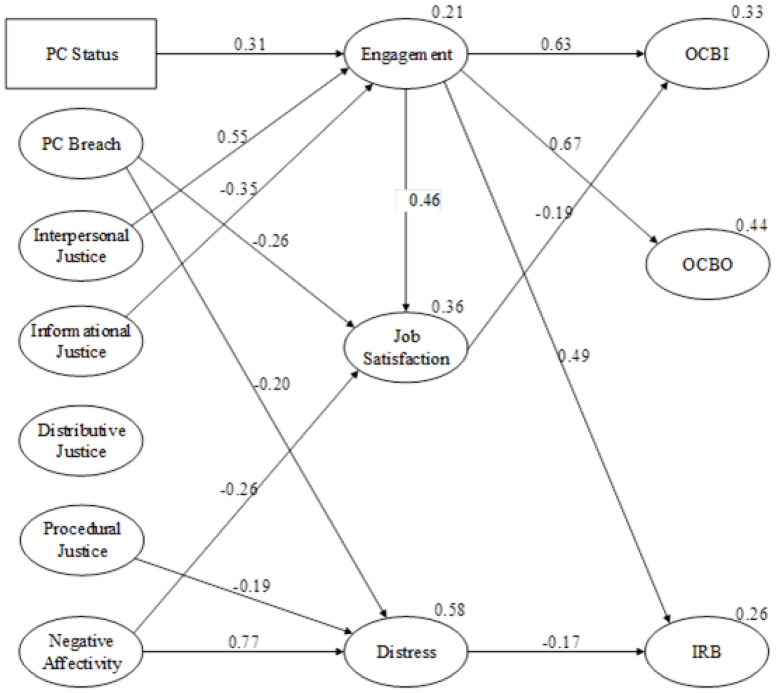
Final structural equation model showing all of the significant relationships.

The test statistics for the initial model (χ^2^ = 1318.78, df = 895, *p* < 0.001, Bollen-Stein *p* = 0.049) and the model after the non-significant paths were removed (χ^2^ = 1349.38, df = 912, *p* < 0.001, Bollen-Stein *p* = 0.046) led to the final model (in Figure 1). Modification indices and standardized residuals indicated that a path from engagement to job satisfaction should be added, resulting in a model with a reasonable fit to the data (χ^2^ = 1307.16, df = 911, *p* < 0.001, Bollen-Stein *p* = 0.076, χ^2^/df = 1.435, SRMR = 0.0599, RMSEA = 0.043 and CFI = 0.95).

Note that in order to enhance the clarity of Figure 1, the correlations between the predictor variables were removed. The variance explained for a variable is indicated above the variable. For example, 0.33 indicates 33% of the variance for OCBI was explained. The standardized weightings for each of the significant relationships are presented near the mid-point of each of the arrows indicating a relationship between variables.

## 4. Discussion

This study examines aspects of the employment relationship (i.e., PC Status, PC Breach and four types of organizational justice) predicting three indicators of performance behaviors (i.e., OCBI, OCBO and IRB). Overall, the results of the current study indicate that engagement, job satisfaction and psychological distress play mediating roles between the psychological contract variables, justice, negative affectivity and types of performance behaviors, representing various mechanisms through which the nurse-employer relationship impacts their performance behaviors, such as motivational and exchange processes.

PC Status was related to the motivational variable of engagement. The impact of PC Breach on the performance behaviors was mediated by attitudinal mechanisms, with job satisfaction reflecting attitudes towards the job and work, and emotional mechanisms as represented by psychological distress, supporting and extending Yeh et al., [7] and Rodwell and Gulyas [10]. The findings suggest that consistent fulfillment of important obligations, represented by PC Status, may keep nurses engaged and consequently performing well, whereas preventing PC Breach may help nurses remain happy in their work.

The positive relationships between PC Status and engagement, and between engagement and OCBI, OCBO and IRB, indicate a key mediating role of engagement between PC Status and the performance behaviors. Moreover, engagement was the most consistent predictor of performance behaviors overall, mediating positive relationships between PC Status, interpersonal justice and OCBI, OCBO and IRB, confirming previous findings of the employee-level performance benefits of engagement for nurses [6] and extending May et al. [29].

That is, benefits that employees have previously received from their employer, reflected in high PC Status, create feelings of obligation to reciprocate efforts by performing beyond what is normally expected [13]. The positive relationship between PC Status and engagement, then connecting to job satisfaction means that PC Status is positively related to job satisfaction through the mediation effects of engagement, rather than direct effects suggested by previous studies [16].

The direction of the relationship between PC Breach and job satisfaction was negative, supporting previous findings [7]. However, the finding that PC Breach negatively impacts distress was unexpected, confirming that distress needs to be included in more psychological contract research (as called for by Brunetto et al. [11], Robbins et al. [17] and Topa and Jurado-Del Pozo [8]). Notably, breach still had these two significant relationships above and beyond negative affectivity. That is, nurses with high negative affectivity had lower job satisfaction and higher psychological distress (following and extending Perez-Fuentes et al. [23]), supporting its inclusion due to the fundamentally perceptual nature of the psychological contract and justice constructs (e.g., [34]). When interpreted in combination the message could be of the nurses being dissatisfied, but not internalizing the breach as distress and instead ‘accepting fate’, possibly as a precursor to withdrawal and eventually leaving the organization.

### 4.1. Organizational Justice

The four types of justice had different relationships to the later variables, confirming that each type is distinct and that all four types should be examined separately (as per Colquitt [18]). The negative link between procedural justice and distress indicates that unfair procedures negatively impact nurses’ mental health, supporting previous research [21]. Nurses may internalize unfair treatment by organizational processes because, following Cohen-Charash and Spector [20], they may feel that the organization was purposefully unfair, or purposefully denying resources [15], resulting in poor affective outcomes such as psychological distress.

Despite theoretical and empirical reasons for expecting distributive justice to impact outcomes such as job satisfaction through perceived inequity [18], there was no significant impact of distributive justice in the current study, a non-relationship also found in Rodwell and Gulyas [10]. One possible explanation for this non-significant finding is that the influence of distributive justice may be obscured by shared variance with PC Breach or PC Status, or, that process aspects of fairness had primacy, rather than outcome fairness.

Interpersonal and informational justice types are often lumped together into interactional justice [18], yet in the current study, interpersonal justice positively related to engagement and, unexpectedly, informational justice negatively related to engagement, demonstrating that they should be separately examined. The finding that interpersonal justice positively relates to engagement supported previous findings [21] and indicating that interpersonal justice (e.g., personal treatment and respect from organizational representatives) may play motivating and enabling roles for nurse engagement, with engagement mediating the relationship between interpersonal justice and performance behaviors.

There may be a range of reasons for the unexpected negative link between informational justice and engagement. For instance, high informational justice may indicate that nurses are overwhelmed by too much information from management to the extent that it distracts them and prevents them from engaging in work tasks by making nurses focus their energy on the information. Another possible explanation is that informational justice may be high primarily in times when organizational representatives feel they need to provide lots of information to ensure they fully explain bad outcomes for nurses (e.g., when nurse bullying occurs [9], whereas during times with less organizational tension and change high levels of information is less important. Thus, high informational justice may happen to coincide with negative system events, rather than causing poor outcomes.

Overall though, with nurses having stepped-up to the challenges from a workforce shortage, worsening demands and a pandemic, the norm of reciprocity that drives the psychological contract suggests that those nurses are owed by their employing organizations. There have been suggestions that the challenges nurses face be recognised tangibly (e.g., increased pay, reducing working hours) and indirectly through increased psychological support [2]. This study’s results also suggest that to address their obligations, employers not only need to implement changes to working conditions, but also need to be seen to be implementing the processes of those changes fairly, for example through the involvement of staff. If organizations start meeting their obligations in efforts to rebalance the state of the psychological contract, then that may slow the otherwise likely withdrawal of nurses from their organizations and potentially the industry. Many of those nurses at risk of departure will be older, more experienced and skilled nurses, who make a large direct impact on health care quality, but also guide more junior nurses [1]. Most directly though, properly managing the state of the psychological contract will enhance the performance of nurses, both in-role and for the extra-role performance behaviors that help organizations to run.

### 4.2. Limitations

In the current study, causality cannot be determined due to the measurement being cross-sectional rather than longitudinal. The current study also relies on self-report measures, though these perceptual measures are appropriate because the focus was on perceptual aspects of the psychological contract and organizational justice. Further, this study explicitly included the main likely cause of any possible self-report or common method bias by considering negative affectivity. Future research needs to strengthen this study’s contribution, by replicating the findings and studying PC Status with longitudinal methods, as well as by widening the scope of issues studied, such as by incorporating patient safety oriented indicators that have been associated with engagement and OCBs.

## 5. Conclusions

High PC Status, representing past successful exchanges, improves the motivation reflected in engagement at work, which then improves in-role and extra-role performance. Enhancing the engagement of nurses is critical to improving both their in-role and discretionary performance. Nurses’ willingness to go the extra mile and perform OCBs has been argued to impact the efficiency and viability of hospitals, especially because nurses have the most frequent interactions with patients and their behaviors strongly influence the quality of care provided [4].

The three more process-related forms of justice also influenced the outcomes, where different justice types reflect aspects of inclusion, information overload, or processes. The current study also highlights the important role that individual differences, in this case negative affectivity, may have. The state of the nurses’ psychological contract acted to motivate and engage both in-role and discretionary performance behaviors. Unless they impacted engagement, breach and certain dimensions of organizational justice tended to act more as brakes on performance behaviors, although the individual’s disposition toward negative affectivity also played a role.

The extent to which high performance levels could be sustained depends on protecting the nurses’ mental health, particularly by not breaking promises, and ensuring key employment processes are fair. Nurse managers can provide an environment where nurses perform their core roles well and are more likely to ‘go the extra mile’, by ensuring that their nurses’ psychological contracts are in a good state and that the managers use their interpersonal skills to ensure processes are fair.

## Figures and Tables

**Table 1:** Means, standard deviations, correlations and Cronbach’s alpha coefficients of the predictor and outcome variables.

Variables	M	SD	1	2	3	4	5	6	7	8	9	10	11	12
1.PC Status	87.18	31.93	--											
2.PC Breach	8.82	3.08	0.30 *	(0.96)										
3.Procedural justice	14.21	4.44	−0.30 *	−0.40 *	(0.88)									
4.Distributive justice	8.98	4.55	−0.28 *	−0.33 *	0.48 *	(0.92)								
5.Interpersonal justice	14.36	4.06	−0.23 *	−0.31 *	0.57 *	0.29 *	(0.95)							
6.Informational justice	12.85	3.96	−0.26 *	−0.35 *	0.62 *	0.43 *	0.72 *	(0.94)						
7.Negative affectivity	8.26	3.69	0.19 *	0.32 *	−0.13 *	−0.16 *	−0.14 *	−0.23 *	(0.83)					
8.Engagement	19.01	2.96	0.23 *	0.01	−0.06	−0.01	0.16 *	−0.05	0.22 *	(0.71)				
9.Job satisfaction	21.48	4.83	−0.09	−0.31 *	0.22 *	0.19 *	0.30 *	0.19 *	−0.22 *	0.34 *	(0.88)			
10.Psychological distress	8.94	3.60	0.09	0.15 *	−0.20 *	−0.16 *	−0.20 *	−0.27 *	0.62 *	0.07	−0.24 *	(0.86)		
11.OCBI	36.48	3.36	0.22 *	0.13 *	−0.04	−0.11	0.12	−0.02	0.14 *	0.42 *	0.07	−0.07	(0.80)	
12.OCBO	43.35	4.12	0.17 *	−0.04	0.15 *	0.07	0.19 *	0.10	0.02	0.46 *	0.22 *	−0.14 *	0.51 *	(0.66)
13.IRB	19.69	1.74	0.15 *	0.02	−0.04	−0.05	0.09	−0.01	−0.02	0.37 *	0.21 *	−0.23 *	0.49 *	0.44 *

Note: PC = Psychological Contract, OCBI = Organizational Citizenship Behavior (Individual), OCBO = Organizational Citizenship Behavior (Organization), IRB = In-Role Performance. Reliabilities are along the diagonal except for IRB, which is 0.97. M = Mean, SD = Standard Deviation. * *p* < 0.05.

## Data Availability

The data are not publicly available due to privacy reasons.
